# The Role of EFL Teachers’ Emotioncy in Preventing Students’ Boredom and Pessimism

**DOI:** 10.3389/fpsyg.2022.880234

**Published:** 2022-05-17

**Authors:** Xiaoshi Zhang

**Affiliations:** Department of Foreign Languages, Shanxi Datong University, Datong, China

**Keywords:** teachers’ emotioncy, student boredom, student pessimism, EFL teacher, positive psychology

## Abstract

It is widely approved that emotions play a critical role in language education. This inspires EFL teachers to establish a classroom oriented toward students’ emotions and senses involved in the teaching/learning processes. Such an emotioncy-based pedagogy can bring about various positive outcomes in second/foreign language education. In tune with this, the present study briefly reviews the definitions, models, roots, and potentials of emotioncy in stopping student’ boredom and pessimism. Moreover, it makes some references to empirical inquiries in this line of research to strengthen its scientific basis. Finally, the study presents a number of implications for teachers, teacher educators, and researchers in language education urging them to focus on students’ emotions and senses more than before.

## Introduction

It is widely accepted that success in language teaching/learning depends on many psycho-emotional factors (Benevene et al., 2020). Due to their critical role, emotions have long been at the core of education irrespective of their valence (negative, positive). In second/foreign language contexts, emotions have surrounded the teachers and students to such an extent that the job is now recognized as one of the most emotionally-tense professions worldwide inundated in emotional traces (Li, 2021; Wang and Derakhshan, 2021). This emotion-based approach to L2 education was positioned by *positive psychology* (PP, hereafter) which highlighted the power of one’s emotions in his/her growth and success (MacIntyre et al., 2019; Mercer, 2020; Wang et al., 2021). In a classroom which is a representation of society, this is emotion and sense that brings teachers and students together and establishes a strong social bond among them (Shayesteh et al., 2019). Aside from teachers’ pedagogical expertise, students mainly learn in a context where their senses are involved in the classroom practices or the emotioncy level is high (Miri and Pishghadam, 2021).

The concept of emotioncy, which is a “mixture of *emotion* and *frequency,”* was first coined by Pishghadam et al. (2013a) to refer to individuals’ unique understanding of the world, concepts, and entities through their emotions and senses. The emotioncy level of people vary in relation to the type, degree of exposure, and sense involvement of a task (Pishghadam et al., 2013a). Basically, this concept was a call for the “emotionalization” of language education by forming an affective/emotional linkage between teachers and students (Pishghadam and Shakeebaee, 2020). When EFL students’ emotions and senses are engaged in an activity or practice, they easily make meaning out of it and find it achievable. In contrast, if the concept/entity carries no sense and does not touch students’ emotional background, they consider it difficult and boring. Research shows that EFL teachers’ emotioncy level influences students’ willingness to communicate (WTC), academic achievement, retention, language competence, reading comprehension, and test performance (Pishghadam, 2016; Pishghadam et al., 2016; Pishghadam and Abbasnejad, 2017; Azamnouri et al., 2020).

Despite these promising outcomes and many other potentials for L2 education, in case emotioncy is ignored or is weak among teachers, dire consequences and impediments emerge. One such problem is student boredom that is recognized by slower perceptions of time and is manifested by non-engagement or departure from activities (Derakhshan et al., 2021a). It is a common emotional state in academia that can hamper students’ classroom performance and achievement (Pawlak et al., 2020; Pawlak et al., 2021). It has long been passed over by other negative emotions like stress and anxiety and EFL teachers considered it trivial (Pawlak et al., 2020). Recently, however, some respectable attempts have been made by scholars on the causes and solutions of boredom in various contexts including, China, and Iran showing the growth of this line of research (Kruk and Zawodniak, 2020; Pawlak et al., 2020; Derakhshan et al., 2021a; Li, 2021). The results of such studies pointed to various sources of student boredom including factors related to students, teachers, tasks, and facilities. Concerning teacher-related factors, monotonous class and flow of instruction was found to be an antecedent of boredom. This monotony can be attributed to EFL teachers’ low emotioncy level and knowledge of emotion-based pedagogy. In a class where the students are bored and their senses are not appreciated by the teacher, other damaging and aversive feelings like pessimism take root in students. Pessimist students have negative moods and resist in the face of challenges. They consider challenge as a threat, so they often have negative self-talks about their future and learning (Yates, 2002). Moreover, they ascribe their failures to fixed and unchangeable events outside themselves. In L2 learning, this negative emotion can ruin all attempts and methodologies if teachers do not take immediate action against it. Nevertheless, scant research has been done on student pessimism and boredom in EFL contexts and the possible role of teacher emotioncy in preventing them. These emotions are related to L2 education in EFL contexts in that learning a foreign language is emotionally tense and generates various emotional reactions among students. However, limited studies have been done on these three constructs at the same time. To fill this gap, this study reviews the definitions, models, studies, and conceptualizations of emotioncy, boredom, and pessimism and offers future research directions.

## Background

### The Concept of Emotioncy

For a long time, the role of emotions and senses in language education and acquisition was neglected until the groundbreaking study of Greenspan (1992) which highlighted a language learning approach based on emotional relationships. Later, this movement motivated Pishghadam et al. (2013b) to invent a term known as “emotioncy” which is combination of emotion and frequency. Based on this concept, each individual has a different emotions and degree of sense involvement when facing a concept/entity. As long as a person’s emotional engagement and frequency of exposure to an input or task increases, he/she learns more and performs better (Pishghadam et al., 2013b). The concept of emotioncy posits that each person has an idiosyncratic way of understanding an entity as their senses determines so. Hence, it is essential to note that the degree of emotioncy can be affected by both internal and external forces as people normally make meaning of things through various sensory channels (Pishghadam et al., 2016). In sum, the notion of emotioncy can be defined as the degree to which EFL classes are emotionalized and there is an inter-emotionality between the students and teachers. It is care for senses that improves the level of academic engagement and performance of EFL teachers and students.

### Models of Emotioncy

For emotioncy, as a novel term in language education, three models have been proposed so far. The first model is a six-level matrix developed by Pishghadam (2015) depicting an individual’s emotioncy level toward an entity. It includes three levels of *avolvement*, *exvolvement*, and *involvement* whose descriptions are provided in Table 1. Simply, avolvement refers to a lack of emotion, exvolvement comprises external emotions, and involvement means being emotionally engaged from inside (Pishghadam et al., 2016).

**TABLE 1:** Types of Emotioncy.

Type	Experience
Null emotioncy	When an individual has not heard about, seen, or experienced an object or a concept
Auditory emotioncy	When an individual has merely heard about a word/concept
Visual emotioncy	When an individual has both heard about and seen the item
Kinesthetic emotioncy	When an individual has touched, worked, or played with the real object
Inner emotioncy	When an individual has directly experienced the word/concept
Arch emotioncy	When an individual has done research to get additional information

It should be noted that in this model, avolvement includes null emotioncy, exvolvement encompasses auditory, visual, and kinesthetic emotioncies, and involvement includes inner and arch emotioncies. These levels work additively in that each stands on the previous one and covers its features. Hence, emotioncy can move forward and backward depending on various factors. The second existing model for emotioncy is a pyramid proposed by Pishghadam (2016). The pyramid includes six layers divided into three macro-categories of avolvement, exvolvement, and involvement. Null emotioncy occurs when there is no emotional involvement or the person has no clue of the entity. Then he/she can move ahead and obtain some audio-visual and kinesthetic inputs from the environment and gets external emotions. At involvement level, the individual may collect all senses and experience the entity as reflected in reality (inner emotioncy) or go further and understand different dimensions/layers of something by conducting research on it (Shahian et al., 2017).

**FIGURE 1:**
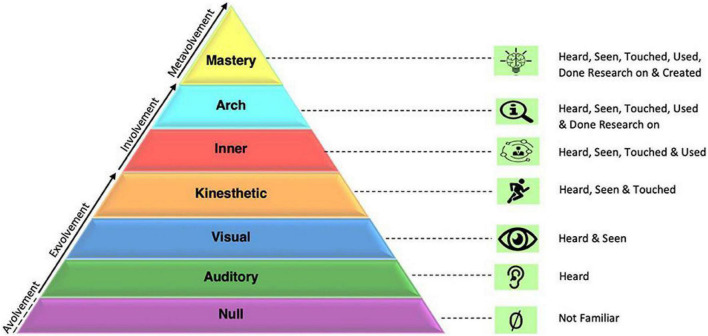
Emotioncy matrix [reproduced with permission from Pishghadam et al. (2019)].

Later, the matrix was extended by Pishghadam et al. (2019) who added metavolvement (mastery) level as the ultimate level of the emotioncy that encompasses the other levels (Figure 1). This model comprises the third model of emotioncy and is meaningful and pertinent to the research objectives of this article in that emotioncy, boredom, and pessimism have been scientifically approved as constructs that are affected by internal and external factors. Hence, when a teacher is at his/her mastery level of emotioncy in which he/she is able to develop and produce content and conduct research, systematic steps can be taken to feed “metavolvement” into students as well to stop or minimize their negative emotions. Since one of the sources of the constitution of students’ boredom and pessimism is teachers’ level of involvement in the educational process, when students see their teachers to be at the “mastery” stage of emotioncy, their degree of boredom and pessimism can reduce, too.

### Emotioncy and Second/Foreign Language Education

The involvement of emotions and senses of L2 teachers and students in teaching, learning, and assessment has been shown to generate numerous positive outcomes as well-documented in PP (Mercer, 2020). Emotion-based education meaningfully improves academic performance, engagement, passion, joy, optimism, hope, resilience, interpersonal communication skills, WTC, test achievement, cooperation, cognition, retention, and many other aspects of L2 education (Pishghadam et al., 2016; MacIntyre et al., 2019; Azamnouri et al., 2020; Wang and Derakhshan, 2021, among others). Other than these positive constructs, emotioncy can decrease and suppress negative feelings such as fear, tension, boredom, pessimism, self-doubt, low self-efficacy, shame, shyness, helplessness, hopelessness and so forth. These ideas signify that emotioncy-oriented education in EFL contexts is a promising opportunity to be attended by L2 practitioners. One of the venues upon which emotioncy and sense-oriented research can shed more light is boredom as a negative feeling that considerably hinders L2 education.

### The Notion of Boredom

Boredom is a multi-dimensional concept that can have different definitions. It is a mixed-emotion of disengagement, dissatisfaction, lack of attention, motivation, vigor, and distorted time perception (Pawlak et al., 2020; Wang et al., 2022). It has long been mixed up with terms such as tedium, sluggishness, indifference, and inactivity. Boredom is a concealable emotion that can stay unnoticed in students unlike anger or anxiety (Pekrun, 2006). Moreover, it involves many socio- psychological and educational factors making unidimensional explanations impossible for the term (Daniels et al., 2015). However, it is widely accepted that boredom is a negative, silent, devastating, temporary, neutralizing, and disappointing emotion that affects learners’ learning (Li, 2021). It prevents students and teachers from enjoying their practices and context and causes disengagement, relentlessness, and fatigue that produce poor academic performance.

### The Typologies and Dimensions of Boredom

Based on durability, boredom can be *trait* or *state* with the former being a stable and frequent disposition experienced when doing a task, while the latter is a momentary sense of boredom in a specific situation (Putwain et al., 2018). Furthermore, according to the degree of un/pleasantness, boredom is divided into *indifferent* (an enjoyable state of fatigue, tranquility, and withdrawal that usually occurs in free time), *calibrating* (a moderately unpleasant feeling with periodic thoughts unrelated to classroom topic, content, and materials), *searching* (a sense of restlessness by which the students seek to change the current situation and look for doing something interesting), *reactant* (a strong negative feeling in students who seek a way to escape the situation, therefore behave belligerently), and lastly *apathetic boredom* (an strong bad state of dissatisfaction and helplessness prevalent in learners with low positive and negative emotions).

Regarding its dimensions, boredom is composed of three dimensions of *valence, activation/arousal*, and *objective focus*. Valence is the extent of pleasantness/unpleasantness of a feeling, activation concerns the physical/cognitive activation/deactivation of an emotion, and finally objective focus concerns whether a feeling is activity-oriented or outcome-oriented (Pekrun, 2006).

### Causes of Boredom

Boredom can appear due to different factors including those related to students, teachers, tasks/materials, and facilities (Li, 2021). Concerning student-related factors, their low language proficiency, low motivation/interest, physical exhaustion, and negative appraisal are the main sources of boredom. Teacher-related causes include their poor teaching skills, untimed feedback, boring speech/presentation, and weak rapport with students. Moreover, task-related causes involve uninteresting, non-engaging, and unchallenging classroom tasks/activities and materials used in the class. Sometimes, insufficient facilities result in disengagement and boredom in both teachers and students. They need digital tools, audio-visual equipment, and appropriate whiteboards and markers as the first requirements of education (Daniels et al., 2015; Derakhshan et al., 2021b; Li, 2021; Wang et al., 2022).

### Pessimism in Education: Causes and Consequences

As stated earlier, teaching and learning are affected by numerous variables and factors. One of such drives is teachers’ and students’ attribution style of how they ascribe the cause of their behavior/action. This generates a frame of reference represented through a continuum of optimism to pessimism (Seligman, 1995). In education, optimist individuals attribute their performance and events to permanent, personal, and ubiquitous causes, while pessimists cast the blame on external and fleeting factors. This negative attribution can cause different problems and setbacks in EFL contexts such as reducing and halting students’ achievement, motivation, and academic engagement, and educational outlook (Yates, 2002). Additionally, when students fringe in the face of challenge and see the future negatively and desperately, they lose their passion and zest for learning and participation as well. Correspondingly, this aversive emotion leads to boredom and tiredness among the students that destroys all methodologies.

Like boredom, students’ pessimism can emerge due to factors related to the student, teacher, and external forces. Students’ personality and genetic features can push students to feel negative about their education and future. Their own worldview and effort is another effective cause. Considering teachers, their personality, instructional expertise, interpersonal communication skills, emotion-oriented pedagogy, rapport, and teaching style are very influential in shaping pessimism. Finally, external circumstances like family problems, illness, job loss, and similar traumatic experiences can also generate students’ pessimism (Riveiro, 2014). As stated, all these problems can be solved *via* an emotioncy-based instruction in EFL contexts. That is to say, an academic milieu wherein teachers and students’ emotions and affective factors are considered can facilitate the development of positive emotions and outcomes and constraint negative emotions. In an emotionally-rich environment, EFL teachers feel better and perform more efficiently leaving no room for negativities and damaging emotions.

### Implications, Research Gaps, and Future Directions

The present review article can have implications for language teachers as well as those of other disciplines in that they can understand the prominence of their care of students’ emotions and senses in the process of teaching and learning. They can also use the ideas of the study in creating proper classroom methodologies and tasks to improve students’ academic interest and engagement *via* involving their pre-existing senses. Moreover, teacher trainers can benefit from this review by proposing training courses in which emotioncy-oriented teaching skills/techniques are taught to EFL teachers along with general teaching requirements. They can design professional development programs in which the conceptualization and operationalization of emotioncy and its potential to stop negative emotions (boredom, pessimism) are explained. Furthermore, L2 researchers can use the ideas put in this study and conduct similar and complementary research on the existing gaps. For example, they can run experimental studies on the efficacy of specific teaching methods on reducing boredom and pessimism. Most studies on students’ boredom and pessimism are in general education/psychology, hence EFL contexts need more investigations in these domains. Future studies can be done on the impact of cultural contexts on teachers’ emotioncy-level to see if EFL teachers from different settings have different conceptualizations of the term. Additionally, mixed-methods, qualitative, and longitudinal studies are recommended to replace one-shot explorations common in this strand of research to capture the developmental trajectories of teachers’ emotioncy and its numerous outcomes. Finally, research is suggested on the potential of emotioncy-based instruction on other PP variables (resilience, love, hope, buoyancy, immediacy, clarity, credibility, academic zest, engagement etc.) and its role in preventing L2 negative emotions (hopelessness, shyness, shame, helplessness etc.).

## Author Contributions

The author confirms being the sole contributor of this work and has approved it for publication.

## Conflict of Interest

The author declares that the research was conducted in the absence of any commercial or financial relationships that could be construed as a potential conflict of interest.

## Publisher’s Note

All claims expressed in this article are solely those of the authors and do not necessarily represent those of their affiliated organizations, or those of the publisher, the editors and the reviewers. Any product that may be evaluated in this article, or claim that may be made by its manufacturer, is not guaranteed or endorsed by the publisher.
